# Conjunctive Analyses of Bulk Segregant Analysis Sequencing and Bulk Segregant RNA Sequencing to Identify Candidate Genes Controlling Spikelet Sterility of Foxtail Millet

**DOI:** 10.3389/fpls.2022.842336

**Published:** 2022-04-14

**Authors:** Yongbin Gao, Lihong Du, Qian Ma, Yuhao Yuan, Jinrong Liu, Hui Song, Baili Feng

**Affiliations:** ^1^State Key Laboratory of Crop Stress Biology for Arid Areas, College of Agronomy, Northwest A&F University, Xianyang, China; ^2^Dexing Township Agro-Pastoral Comprehensive Service Center, Nyingchi, China; ^3^Anyang Academy of Agricultural Sciences, Anyang, China

**Keywords:** foxtail millet, spikelet, sterility, BSA-seq, BSR-seq

## Abstract

Foxtail millet has gradually become a model gramineous C_4_ crop owing to its short growth period and small genome. Research on the development of its spikelets is not only directly related to the yield and economic value of foxtail millet but also can provide a reference for studying the fertility of other C_4_ crops. In this study, a hybrid population containing 200 offspring was constructed from the Xinong8852 and An15 parental lines, and two extreme trait populations were constructed from the F2 generation for analysis of the spikelet sterility. The F2 population conformed to a 3:1 Mendelian segregation ratio, and it was thus concluded that this trait is likely controlled by a single recessive gene. Bulk segregant analysis sequencing (BSA-Seq) was used to determine the candidate regions and candidate genes related to the development of foxtail millet spikelets. Additionally, the functional analysis of differentially expressed genes in populations with different traits was conducted by bulk segregant RNA sequencing (BSR-Seq). Finally, conjunctive analysis of BSA-Seq and BSR-Seq results, combined with biological information analysis, revealed six genes on chromosome VII that were ultimately identified as candidate genes controlling foxtail millet spikelet development. This study provides a new reference for research on foxtail millet sterility and lays a solid foundation for the examination of fertility in other gramineous crops.

## Introduction

Foxtail millet originated in China and has a cultivation history of more than 10,000 years. It has high nutritional value because of its dual uses as food and animal feed. At the same time, because of its short growth period, wide adaptability, drought tolerance, and tolerance of barren land, foxtail millet is widely cultivated in temperate and tropical arid and semiarid regions of Eurasia, where it plays a vital role in ensuring regional food security (Shan et al., 2014; Verma et al., 2015). With the completion of whole-genome sequencing of foxtail millet (Pandian and Ramesh, 2019), it has gradually become a model gramineous C_4_ crop, as well as a model for studying drought and stress tolerance, owing to its small diploid genome, self-pollination, high reproductive coefficient, small plant height, simple cultivation methods, short growth cycle, and easy, rapid propagation (Doust et al., 2009; Jia and Diao, 2017; Yang et al., 2020).

The development of the floral organs of crops is directly related to crop fertility, and it is also the basis for the yield formation and economic value of crops. Their morphogenesis and development are affected by the environment and involve complex genetic regulatory networks (Wu and Xu, 2009). Researchers have studied the growth and development of floral organs in rice (Jeon et al., 2000), maize (Yi et al., 2021), and wheat (Debernardi et al., 2020) previously, and also established the classical ABC, quartet, and floral organ characteristic gene activity models (Kaufmann et al., 2005; Soltis et al., 2007; Wu and Xu, 2009), which have further revealed the molecular mechanism of floral organ development in gramineous crops. In recent years, the key genes involved in top spike abortion (Li et al., 2015; Xue et al., 2018), anther color (Han et al., 2019), and male sterility (Yang, 2013) have also been reported successively. However, few studies have examined the genes related to the spikelet of foxtail millet. Further exploration of spikelet development in foxtail millet will further clarify the mechanisms of foxtail millet fertility and provide new opportunities for fertility research on other C_4_ crops, as well as for the utilization of foxtail millet heterosis.

Bulked segregant analysis sequencing (BSA-Seq) can be used to detect the allele frequency (AF) of single-nucleotide polymorphisms (SNPs) by constructing the DNA pool of offspring with extreme traits and using molecular markers to perform co-segregation analysis between markers and traits in the two pools. Thus, BSA-Seq enables the rapid identification of genes associated with qualitative traits controlled by a single gene or major genes associated with quantitative traits. Bulked segregant RNA sequencing (BSR-Seq) is the combination of BSA-Seq and RNA-Seq, and it has been utilized in the localization of the maize yellowing mutation gene (Liu et al., 2012), the localization of the wheat powdery mildew resistance gene PmSESY, and the study of maize male sterility (Su et al., 2016). Similarly, de Lima Castro et al. (2017) localized a novel locus in Andean bean that is associated with anthracnose resistance by using BSA-Seq. Li et al. (2019) found the casein kinase gene BrCKL8, a key gene for forming Chinese cabbage leaf heads, by using BSA and RNA-Seq combined analysis, revealing the critical pathway of head formation in this crop.

In this study, we used the Xinong8852 and An15 parental lines of foxtail millet to construct a single F2 population that contained 200 individual plants. After field investigation, the traits related to the fertility of the F2 population conformed to a 3:1 Mendelian segregation ratio, and it was thus concluded that this trait is likely controlled by a single recessive gene. Then, BSA-Seq was used to sequence the two populations with extreme traits, which revealed the AF of SNPs from the two populations. This was followed by a correlation analysis based on the differentially expressed genes (DEGs) obtained by BSR-Seq analysis. Thus, this study aimed to localize infertility-related genes and loci and analyze the molecular mechanism of floral organ sterility in foxtail millet.

## Materials and Methods

### Planting and Selection of Test Materials

In our study, Xinong8852 (XAP1) and An15 (AYP1), as well as the F2 population constructed by their cross, were used as experimental materials and planted in the Crop Teaching Sample Area of Northwest A&F University (Yangling, China). The parents and F2 progeny were planted separately by dibble seeding. At the flowering stage of foxtail millet, the traits were observed, and one parent plant was marked. In the F2 generation, 30 plants with normal spikelet development (KY) and 30 with abnormal spikelet development (BY) were marked. After labeling, fresh leaves were selected from the parents and the extreme population at the same time and stored at –80°C for subsequent DNA extraction for BSA-Seq. Before reaching full bloom, samples were taken from the spikes, respectively, and stored at –80°C for subsequent RNA extraction for BSR-Seq.

### Bulked Segregant Analysis Sequencing

#### DNA Extraction, Library Construction, and Sequencing

The modified cetyltrimethyl ammonium bromide (CTAB) method (Doyle, 1990) was used to extract DNA from fresh leaves from parental materials (AYP1 and XAP1) and extreme individuals in the F2 population. Two parental bulk samples were constructed from the DNA of the parents, and the KY and BY bulk samples were constructed by mixing the DNA of the progeny of normal and abnormal plants in the F2 generation, respectively. The total amount of DNA was detected using a NanoDrop instrument (OD260/280; NanoDrop, Wilmington, DE, United States), and the integrity and concentration of DNA samples were identified using Qubit Fluorometer (Thermo Fisher Scientific, Waltham, MA, United States) and agarose gel electrophoresis. High-quality samples were thus identified and sequenced by Beijing Ovison Gene Technology Co. Ltd. (Beijing, China) using an Illumina high-throughput sequencing platform (NovaSeq 6000; Illumina, San Diego, CA, United States).

#### Detection and Annotation of Single-Nucleotide Polymorphisms

After filtering the raw reads obtained by sequencing to remove the adapter sequences, BWA software (Li, 2013) was used to compare the obtained clean reads with the reference genome, and SAMtools (Li et al., 2009) was used to compare the results to sort and remove duplicates. After SNP detection using the Unified Genotyper model in GATK3.7, variant filtration was used to filter out the sites with SNP index values less than 0.3 and SNP depth values less than 7 in the bulked samples from offspring with extreme phenotypes. ANNOVAR software was used to annotate the filtered SNP detection results, and finally, the high-quality SNPs site set was obtained.

#### Screening and Functional Analysis of Target Genes

Single-nucleotide polymorphism index values were used to represent the sequence difference between the offspring population and their parents, and the SNP index value of each variant locus after quality control in the KY and BY bulk samples was calculated. The loci with SNP index values less than 0.3 and SNP depths less than 7 in the KY and BY bulk samples were filtered, and the homozygous and non-missing SNP loci were then retained. At the same time, the differences between the SNP index values of the two progeny pools were calculated to obtain the delta (SNP index) values. After 1,000 permutation tests, 95% CI were applied as the screening threshold to determine candidate sites. Additionally, functional annotation and analysis of genes corresponding to candidate sites were performed.

### Bulked Segregant RNA Sequencing

#### RNA Extraction, Library Establishment, and Sequencing

The RNA of the two parents and two bulk samples of offspring with extreme phenotypes were extracted using the Trizol method. The concentration and purity of the extracted RNA were detected using the NanoDrop instrument (OD260/280, OD260/230), and the integrity of RNA fragments was detected using the Agilent 2100 platform (Agilent Technologies, Santa Clara, CA, United States). Thus, four bulk samples were obtained for BSA-Seq. Then, after quality control, Beijing Ovison Gene Technology Co. Ltd. completed the sample database construction and sequencing using an Illumina high-throughput sequencing platform (Hiseqtm2500/4000, Illumina). FastQC (version 0.11.9) was used to evaluate the quality of the sequence data. The original data was filtered using Fastp software (version 0.20.0) to remove adapters, uncalled bases, and low-quality sequences to obtain high-quality data (Chen et al., 2018).

#### Read Mapping and Differential Expression Analysis

The genome of the foxtail millet line Yugu1 was used as the reference genome, and the filtered sequencing data were compared using Star software (Dobin et al., 2012). The gene expression level was assessed based on fragments per kilobase of transcript per million fragments mapped (FPKM) values, and the gene expression levels of each sample were analyzed using HTSeq software (Simon et al., 2015). After obtaining the gene expression matrix, read counts were normalized, and the corresponding *p*-values were calculated based on the negative binomial (Pascal) distribution. Finally, multiple hypothesis testing correction (*via* the Benjamini–Hochberg procedure) was performed to control the false discovery rate. DESeq2 (Love et al., 2014) was used to identify DEGs between BY and KY samples, BY and AYP1 samples, and BY and XAP1 samples, respectively. Finally, the DEGs were further screened, and the genes with consistent trends and significant differences between BY and KY and their parents were identified for functional analysis.

#### Functional Analysis of Differentially Expressed Genes

Gene Ontology (GO^1^) is an internationally standardized gene function classification system that can be used to classify DEGs according to their molecular function, biological process, and cell composition (cellular component) ontologies, respectively. We used GOseq software (Young et al., 2010) to perform GO annotation and enrichment analysis of DEGs. The Kyoto Encyclopedia of Genes and Genomes (KEGG^2^) not only comprises all characterized metabolic pathways but also comprehensively annotates the enzymes that catalyze each step of such reactions, including amino acid sequences and PDB library links (Kanehisa et al., 2008), among other features. To further explore the biological pathways associated with DEGs, we first used KEGG automatic annotation server (KAAS) to complete the KEGG pathway annotation of DEGs and obtained the KEGG background file for each whole foxtail millet gene. Then, we used the KEGG Enrichment function in TBtools (Chen et al., 2018) for enrichment analysis.

### Bulk Segregant Analysis Sequencing and Bulk Segregant RNA Sequencing Association Analysis

The candidate loci identified by BSA-Seq were jointly compared with DEGs obtained from the BSR-Seq screen to identify the genes located in variant candidate regions and differentially expressed. Using databases such as the National Center for Biotechnology Information, combined with GO and KEGG annotations, further analysis of the functions and pathways of the selected candidate genes were used to determine the main genes that cause infertility in foxtail millet spikelets and analyze the molecular mechanism of this phenotype.

### Real-Time Quantitative PCR Validation

In order to further verify the accuracy of the above experimental results and the expression of candidate genes in foxtail millet corresponding with differences in fertility, quantitative reverse transcription PCR (RT-qPCR) experiments were conducted using RNA extracted from the panicles of millet in the two progeny pools before flowering. Each sample (100 mg, three biological replicates) was used for the extraction of total RNA. RNA samples were treated with DNase I (New England Biolabs, Ipswich, MA, United States) to remove genomic DNA contamination, followed by cleanup using the GeneJET RNA Cleanup and Concentration Micro Kit (Thermo Fisher, Waltham, MA, United States). A NanoDrop2000 spectrophotometer (Thermo Fisher) and 1% agarose gel electrophoresis were used to evaluate the total RNA concentration and quality of each sample. The validated RNA was uniformly mixed to form the BY sample (from the BY pool) and KY sample (from the KY pool). The cDNA was then prepared from 1 μg of total RNA using All-in-one First-Strand cDNA Synthesis SuperMix (Invitrogen, Beijing, China), following the manufacturer’s instructions, for RT-qPCR analysis. Actin was used as an internal reference gene, and all primers were designed with Primer 5 (Supplementary Table 1). All RT-qPCR assays were performed using Top SYBR^®^ Premix Ex Taq™ II (Tli RNaseH Plus) (Tli RNaseH Plus; Takara, Dalian, China) *via* the Applied Biosystems ABI 7500 Fast real-time PCR machine (Applied Biosystems, Foster City, CA, United States). The cycling conditions were 95°C for 1 min, 40 cycles at 95°C for 15 s, and 60°C for 15 s. Each target gene had three technical replicates, and the relative expression level of the target gene was calculated by the 2^–ΔΔCt^ method.

## Results

### Spikelet Sterility Field Measurements

**FIGURE 1:**
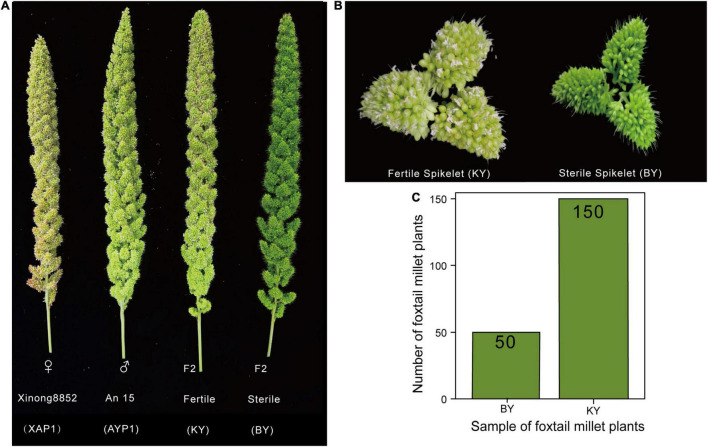
The spike development of two parents and two F2 populations. **(A,B)** The spike and spikelet development of two parents and two F2 populations. **(C)** The number of plants with sterile spikelets and normal spikelets in the F2 population.

The ear growth and development of parents and 200 F2 plants were observed and recorded after the jointing stage of foxtail millet. We found that the spikelets of the two parents could develop normally, with KY plants having no significant difference from their parents in the development of spikelets and panicles, but for BY plants, the glume remained closed and the spikelet could not normally open (Figures 1A,B). After maturity, we investigated the fertility and main agronomic traits of 200 F2 plants (Supplementary Table 2) and found 150 normal spikelets and 50 abnormal spikelets (Figure 1C). A χ^2^ test of the F2 survey results confirmed that the fertility segregation rate of the F2 generation was consistent with a 3:1 ratio (fertile:sterile, χ^2^ < χ^2^_0.05_ = 3.84, *p* < 0.05, Supplementary Table 3), and thus complied with Mendel’s law of segregation for a single recessive allele. Thus, we speculate that this phenomenon may be mainly controlled by a locus with a pair of recessive and dominant alleles (Guan et al., 2016; Han et al., 2019) and preliminarily define this phenotype as infertility of floral organs caused by abnormal spikelet development. To further reveal the mechanism of the phenotype and locate the key genes, we conducted further analysis at the molecular level through a combination of BSA-Seq and BSR-Seq.

### Bulked Segregant Analysis Sequencing

#### Sequencing Quality Assessment and Mapping Analysis

To determine which genetic differences led to the occurrence of foxtail millet spikelet sterility, we used high-throughput sequencing technology to perform whole-genome resequencing of four samples from two parents and two progeny pools with extreme traits; thus, 41,422,537,500 bp of raw data were generated in total. After quality control, 41,214,112,800 bp high-quality clean reads were obtained. The guanine cytosine (GC) content of these reads was 46.31–46.94%, and the quality of the sequencing data was high (Q20 ≥ 97.82%, Q30 ≥ 93.95%) (Supplementary Table 4). Next, we mapped the clean reads obtained from the four samples after quality control based on the reference genome, obtaining a total of 271,043,339 reads. The mapping results showed that the sample comparison rate was between 97.91 and 99.07%, with an average sequencing depth of parents of 10×, and the offspring pools exhibited approximately 30× depth; the 1× and 4× coverage percentages were 95.49 and 90.82, respectively (Table 1). The mapping data shows that the reference genome was evenly and randomly covered, which was conducive to the filtering and screening of SNPs in the next step.

**TABLE 1:** Quality statistics of mapping with the reference genome for BSA-Seq.

Sample	Mapped reads	Total reads	Mapping rate (%)	Average depth (×)	Coverage 1 × (%)	Coverage 4 × (%)
XAP1	36,115,655	36,886,738	97.91	10.57	95.49	90.82
AYP1	32,975,779	33,284,924	99.07	9.94	96.5	91.38
BY	109,123,265	110,367,772	98.87	29.19	98.42	97.76
KY	92,828,640	94,221,318	98.52	26.55	98.42	97.68

*Mapped Reads: the total number of reads on the reference genome was compared.*

*Total reads: the total reads of valid sequencing data.*

*Mapping rate: the number of reads on the reference genome was compared by the number of reads in the valid sequencing data.*

*Average depth: the average sequencing depth, the total number of bases compared to the reference genome divided by genome size.*

*Coverage 1×: the proportion of bases whose coverage depth is not less than 1× in the whole genome.*

*Coverage 4×: the base coverage depth in the whole genome is no less than the base ratio of 4×.*

#### Detection and Annotation of Single-Nucleotide Polymorphisms

ANNOVAR is an efficient software tool that can use the latest information to perform functional annotations of genetic variants detected from multiple genomes. We used it to annotate the functions of detected SNP. By screening the SNPs detected by the unified genotype model of gatk3.7 software, 1,945,742 SNPs were obtained. By further annotating the SNPs with ANNOVAR, it was found that 73,739 SNPs were located in the exon region, including “stop gain” mutations, which cause a full coding sequence gene to contain a stop codon; “stop-loss” mutations, which cause a gene to lose its stop codon; “synonymous” mutations, which do not affect the amino acid sequence; and “non-synonymous” mutations, which alter the amino acid sequence.

#### Location of Candidate Regions and Screening of Genes

**FIGURE 2:**
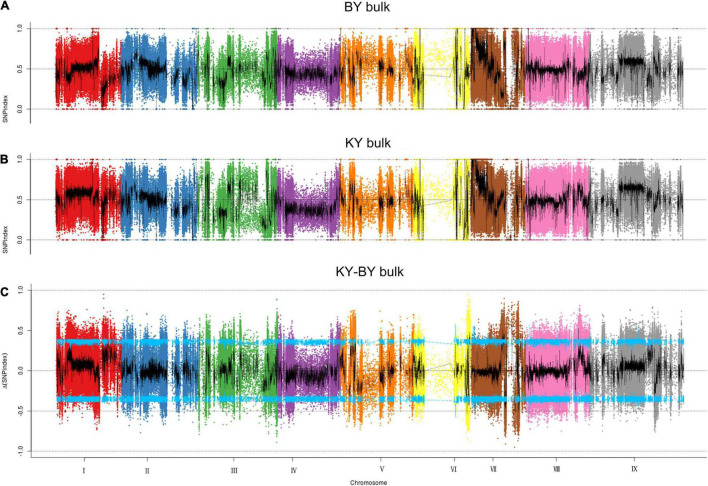
Distribution of SNP index association values on chromosomes. **(A)** Distribution of single -nucleotide polymorphism (SNP) index values of BY bulk on chromosomes. **(B)** Distribution of SNP index values of KY bulk on chromosomes. **(C)** Distribution of Δ(SNP index) value on chromosomes, where the blue line represents the 95% CI.

To determine the mutation sites that exist in the BY bulk sample of foxtail millet, the SNP index method was used to analyze the detected SNPs. First, after filtering out the heterozygous SNPs from the filial generation, the SNP index was less than 0.3, with a depth less than 7, and the sites in each F2 pool lacked SNPs; we identified 926,529 such SNPs from 1,945,742 SNPs. Then, we found a total of 9,055 mutation sites outside the CI by calculating the delta SNP index in the KY and BY bulk samples and screening with a 95% CI (Figure 2), and the annotation of these sites indicated that 523 were located in exons, including mutations that caused the gene to gain or lose its stop codon.

In considering the polymorphisms associated with the occurrence of sterility, we filtered out the sites with synonymous mutations in 523 loci and obtained 3 regions and 211 polymorphisms sites related to sterility from chromosomes III, VI, and VII. By associating the location information of these 211 polymorphic sites with genome annotation information, we identified 90 candidate genes, 70 of which were located between 20,557,247 bp and 31,479,141 bp on chromosome VII, 19 of which were located between 33,984,600 bp and 35,805,805 bp on chromosome VI, and a single candidate gene located between 4,908,634 and 4,912,328 on chromosome III. To further analyze the subcellular localization, molecular function, and biological processes associated with these candidate genes, we performed GO annotation and enrichment analysis for 90 candidate genes. All genes were annotated with 705 GO terms, of which 54 genes were significantly enriched within 23 functional groups, including 13 biological processes, 3 cellular components, and 7 molecular functions. Among the biological processes, these genes are mainly involved in the reproduction process, the development of seeds and fruits, the development of the reproductive system, and the reproductive structure. Among the cellular components, these genes are mainly enriched in the components of the cell membrane. Lastly, among molecular functions, these genes are mainly involved in functions such as binding and catalytic activity.

### Bulked Segregant RNA-Seq Analysis

#### Sequencing Data Analysis of Four cDNA Bulk Samples

In order to further clarify the transcription process associated with organ sterility in foxtail millet, we used the two parents and two extreme trait groups to construct four pools. After establishing three replicates in each bulked pool, we performed RNA sequencing of 12 cDNA libraries on an Illumina (HiSeq™2500/4000) high-throughput sequencing platform. After reads with low quality and adapter sequences were removed, we obtained 187,104,828 clean reads from 193,102,338 raw reads from four samples (Supplementary Table 5). Then, the obtained clean reads were mapped to the reference genome of Yugu1 foxtail millet. The comparison revealed that the unique mapping degree reads rate was between 90.66 and 92.81% (Table 2).

**TABLE 2:** Quality statistics of mapping with the reference genome for BSR-Seq.

Sample name	Total reads	Total mapping rate(%)	Multiple mapping (%)	Uniquely mapping (%)	Reads mapping to + (%)	Reads mapping to – (%)	Non-splice reads (%)	Splice reads (%)
XAP1	48,009,869	93.03	2.36	90.66	45.33	45.33	57.83	32.84
AYP1	47,835,947	94.76	1.96	92.81	46.41	46.41	61.74	31.06
KY	43,620,057	94.70	2.10	92.61	46.30	46.30	60.96	31.64
BY	47,638,955	93.79	3.23	90.57	45.28	45.28	57.76	32.81

*Total reads: the total reads after quality control.*

*Total mapping rate: the rate of total reads that can be mapped to the reference sequence.*

*Multiple mapping: the reads that mapped to multiple positions in the reference sequence. Uniquely mapping: the reads that maps to a unique position in the reference sequence.*

*Reads mapping to +, reads mapping to –: the reads mapped to positive and negative chains, respectively.*

*Non-splice reads: the reads that mapped to only one exon. Splice reads: the same reads section mapped to different exons.*

#### Screening of Differentially Expressed Genes Between Different Bulk Samples

**FIGURE 3:**
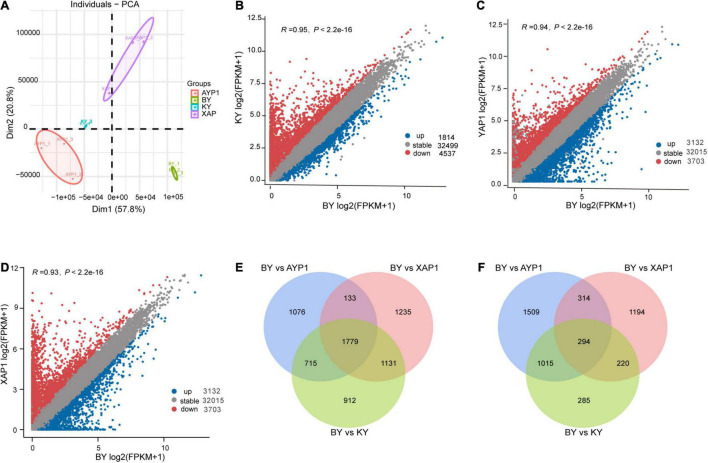
Differences in gene expression among four different samples from two parents and two progeny pools. **(A)** Principal component analysis of four samples, and each sample contains three biological repeats. **(B–D)** The number of differential genes between different samples. Comparison of the expression of gene between BY with KY, BY with AYP1, BY with XAP1 samples. **(E)** The significantly downregulated genes in BY samples compared with other samples. **(F)** The significantly upregulated genes in BY samples compared with other samples.

Through the principal component analysis of RNA-Seq data, we learned that all samples could be clustered by dimensionality reduction, which indicates that the repeatability between the groups of samples was good and that there was a big difference between the samples (Figure 3A). Based on this result, difference analysis was performed using the DESeq2 R package, and genes meeting thresholds of *p*-value > 0.05 and | log_2_(fold change)| > 1 were selected as the DEGs. Spikelet sterility only appeared in the BY sample, but both parents and the KY sample showed normal spikelet phenotypes. We then compared the samples with abnormal floral organs with other samples with normal development to obtain genes related to floral organ sterility in foxtail millet. By comparing differential gene expression between BY with KY, BY with AYP1, and BY with XAP1, we obtained 1,814, 3,132, and 2,022 upregulated DEGs, and 4,537, 3,703 and 4,278 downregulated DEGs, respectively (Figures 3B–D).

Next, we looked for overlap among the three combinations and found their shared DEGs that were upregulated or downregulated for identification as candidate genes related to this study for further analysis. During this process, we obtained a total of 294 shared upregulated DEGs (Figure 3E) and 1,779 shared downregulated DEGs (Figure 3F).

#### Gene Ontology and Kyoto Encyclopedia of Genes and Genomes Pathway Enrichment Analysis of Differentially Expressed Genes

To examine the molecular mechanism of foxtail millet spikelet development, we further analyzed 1,779 downregulated DEGs and 294 upregulated DEGs that were differentially expressed in BY samples compared with other samples.

**FIGURE 4:**
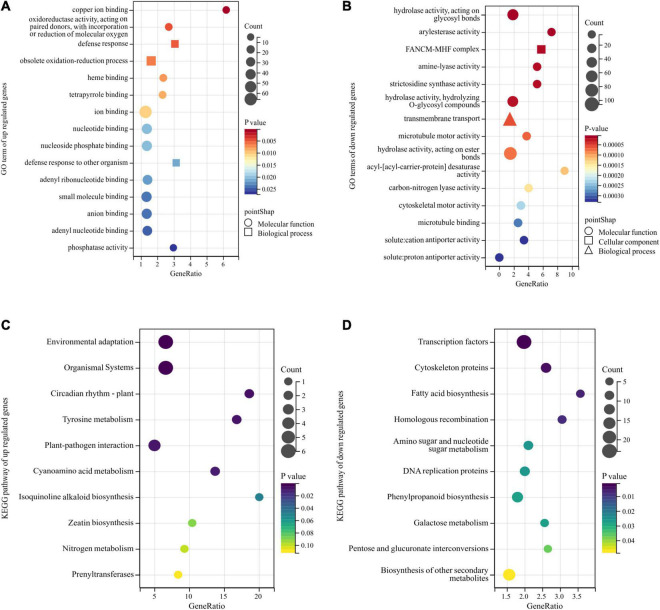
Gene Ontology (GO) and Kyoto Encyclopedia of Genes and Genomes (KEGG) enrichment analyses of DEGs obtained by bulk segregant RNA sequencing (BSR-Seq). Among them, different colors represent the size of the *p*-value, the circular size represents the number of enriched genes, and different shapes represent different functional classifications. **(A,B)** The top 15 GO terms of upregulated and downregulated DEGs enrichment in GO enrichment analysis. **(C,D)** The top 10 pathways of upregulated and downregulated DEGs enrichment in KEGG enrichment analysis.

The GO annotation was applied to the obtained DEGs, and the enrichment analysis was performed with a *p*-value < 0.05 threshold. The genes with relatively high expression in the BY sample were enriched for 25 GO terms, but those with relatively low expression were enriched for 112 GO terms. Among them, the relatively highly expressed genes in samples with abnormal floret development were mainly enriched for the following terms: ion binding, oxidoreductase activity, heme binding, tetrapyrrole binding, nucleotide binding, nucleoside phosphate binding, and adenyl ribonucleotide binding, small molecule binding, anion binding, adenyl nucleotide binding, phosphatase activity, participation in defense response, obsolete oxidation-reduction process, and defense response to other organisms (Figure 4A). The relatively low-expression genes were mainly enriched for cell components, such as FANCM-MHF complex, with hydrolase, arylesterase, amine-lyase, strictosidine synthase, microtubule motor, acyl- [acyl-carrier-protein] desaturase, carbon-nitrogen lyase, and cytoskeletal motor, cation antiporter, and proton antiporter and other activities, and involved in biological processes, such as transmembrane transport and microtubule-based movement (Figure 4B).

Next, KEGG analysis was used for pathway enrichment analysis to analyze the metabolic pathways associated with these DEGs. The genes significantly upregulated in the BY samples were mainly enriched for the pathways of environmental adaptation, organismal systems, plant circadian rhythm, tyrosine metabolism, and plant-pathogen interaction (Figure 4C). The downregulated genes were associated with cyanoamino acid metabolism and isoquinoline alkaloids biosynthesis but were mainly enriched for the transcription factors, cytoskeleton proteins, fatty acid biosynthesis, biosynthesis of secondary metabolites, and homologous recombination pathways (Figure 4D).

The GO and KEGG enrichment analyses of the function and metabolic pathways of DEGs between samples with normal and abnormal spikelet development indicate that the development of foxtail millet spikelet is affected by multiple genes and multiple metabolic pathways.

### Combined Analysis of Bulk Segregant Analysis Sequencing and Bulk Segregant RNA Sequencing

To further explore the main genes controlling spikelet abortion in foxtail millet and explore their function, the BSA-Seq and BSR-Seq results were analyzed jointly. Thus, we found that six candidate genes with mutations on chromosome VII were significantly differentially expressed between the samples with normal spikelet development and the samples with abnormal spikelet development, which included four downregulated genes and two upregulated genes (Table 3).

**TABLE 3:** Basic information of candidate genes.

Gene ID	Gene name	Chromosome position	Gene expression (up or down)
Setit009896mg	Beta-Glucosidase 12	ChrVII: 22,678,921–22,683,049	Up
Setit009293mg	Non-specific serine/threonine protein kinase	ChrVII: 30,845,482–30,849,804	Up
Setit009865mg	Sugar transport protein MST1	ChrVII: 21,688,075–21,691,295	Down
Setit011801mg	VQ motif-containing protein 11	ChrVII: 31,441,080–31,442,616	Down
Setit009909mg	ETHYLENE INSENSITIVE 3-like 5 protein	ChrVII: 21,771,669–21,773,573	Down
Setit011746mg	FBD domain-containing protein	ChrVII: 30,133,024–30,135,230	Down

**FIGURE 5:**
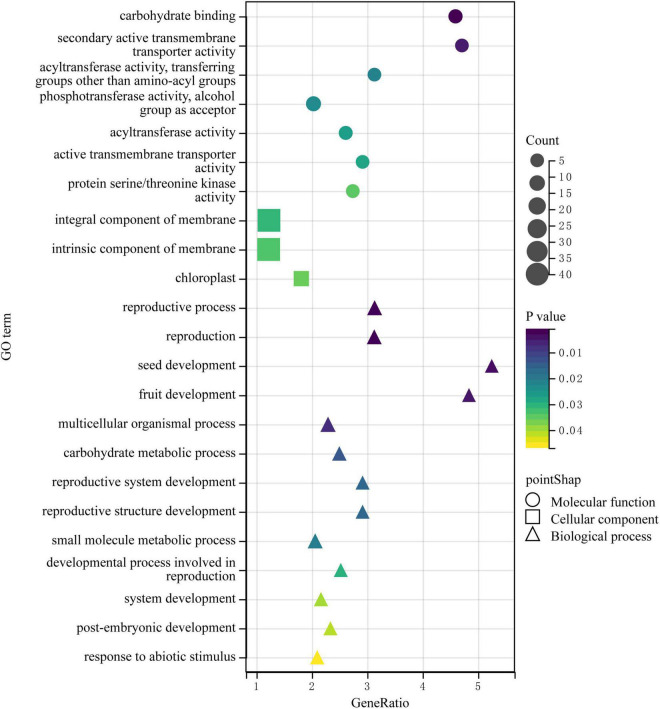
The GO enrichment analysis of DEGs obtained by associating BSA-Seq and BSR-Seq. Among them, different colors represent the size of the *p*-value, the circular size represents the number of enriched genes, and different shapes represent different functional classifications.

Then, we downloaded the gene sequence from NCBI and blasted it against the UniProt database to obtain the basic information on the six genes. Then, GO enrichment analysis was performed on the six obtained genes (Figure 5). Among them, the upregulated gene Setit009896mg was mainly related to hydrolase activity and participated in the metabolism of organic matter and carbohydrates. Additionally, among the significantly downregulated genes, Setit011801mg functions in actin-binding, cytoskeletal protein binding, and protein binding and participates in cellular component organization or biogenesis, cytoskeleton organization, and organelle organization. Setit009865mg is mainly related to transmembrane transport, and it is involved in transmembrane transport and the establishment of localization processes. Setit009909mg functions as a DNA-binding transcription factor and participates in the regulation of the transcription process.

In particular, the upregulated gene Setit009293mg was significantly enriched in biological processes and molecular functions and participates in pollination, pollen-pistil interaction, pollen recognition, pollen recognition, and protein serine/threonine kinase activity. In addition, the downregulated gene Setit011746mg was significantly enriched in biological processes and molecular functions, including rRNA methyltransferase activity, catalytic rRNA activity, RNA methyltransferase activity, and *S*-adenosylmethane-dependent methyltransferase activity, and participates in rRNA metabolic process, rRNA processing, and ribosome biogenesis.

### Validation of Quantitative Reverse Transcription PCR of Candidate Genes Related to Millet Fertility

The expression patterns of the six candidate genes in foxtail millet with different fertility phenotypes were identified by RT-qPCR, which included a glucosidase activity gene (Setit009896mg), a protein phosphorylation gene (Setit009293mg), a gene related to sugar transport (Setit009865mg), a serine/threonine-protein phosphatase PP2A-1 catalytic subunit gene (Setit011746mg), a gene related to DNA binding factor (Setit011801mg), and a gene related to ethylene (Setit009909mg).

**FIGURE 6:**
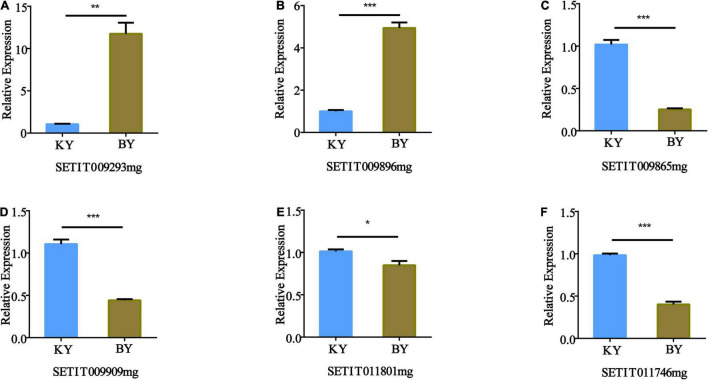
Quantitative RT-PCR (RT-qPCR) validation of the expression of genes identified by BSA-Seq and BSR-Seq. The actin gene was used as an internal control. The transcription level of the spikes of the normal fertility sample (KY) was set at 1.0. Asterisks indicate significant differences at various thresholds (*p < 0.05, **p < 0.01, ***p < 0.001). Error bars represent the mean SE of three biological replicates. **(A–F)** Represent the relative expression of genes in different fertility samples.

Quantitative RT-PCR analysis showed that the expression of Setit009293mg (Figure 6A) and Setit009896mg (Figure 6B) in infertile samples was significantly higher than that in fertile samples (*p* < 0.001). However, the expression levels of Setit009865mg, Setit009909mg, and Setit011746mg were significantly lower than those of fertile samples (*p* < 0.001) (Figures 6C,D,F), and the expression of Setit011801mg was significantly lower than that of fertile samples (*p* < 0.05) (Figure 6E). Thus, the candidate genes’ expression differences between samples were consistent with the BSR-Seq results, which supported our sequencing results.

## Discussion

In most crops, flower development directly affects its economic value. However, when crops are affected by the external environment or have various mutations, infertility is often a consequence. A thorough study of crop infertility and exploring its occurrence mechanism is not only important for improving crop yields and economic value but also for artificial interference breeding and the effective utilization of crop heterosis. Numerous studies have shown that the development of crop spikelets is a precise and complex regulated process, and any factor change in the process may lead to infertility (Litt and Kramer, 2010).

The development of spikelets in the panicle is one of the important characteristics of the development of foxtail millet from vegetative growth to reproductive growth, and it has a direct impact on the yield and quality of foxtail millet. Research on spikelet sterility in foxtail millet mainly focuses on male sterility. In 1967, the Yan male-sterile line was selected from a foxtail millet variety by the Yan’an Agricultural Science Institute (Li, 1997). In 1976, a dominant genic male-sterile line of foxtail millet was first discovered by the Chifeng Institute by Agricultural Sciences in Inner Mongolia from the F3 hybrid between Australian foxtail millet and Turpan foxtail millet. It was systematically determined that the genetic mechanism is the male-sterile gene Ms*^ch^* in foxtail millet, and the existence of multiple alleles causing male sterility in foxtail millet was further confirmed at the level of cytogenetics (Hu et al., 1986; Ma et al., 1992). Li et al. (2010) tested and crossed 10 North China foxtail millet varieties with two high male-sterile lines (117A and Gao146A) and speculated that the sterility of these two sterile lines was controlled by one pair of major recessive genes and also affected by minor polygenes according to the fertility of F2 and BC1F1 segregation populations. Wang et al. (2007) conducted chromosome mapping of genes with 1066A sterile traits, which indicated that genes controlling this sterile trait are located on chromosome VI and recessive. With the continuous progress of research, more studies have shown that foxtail millet chromosome VI has genes related to fertility, especially male sterility (Hao et al., 2011; Jun et al., 2013; Jia et al., 2018). In recent years, the application of some new biotechnology in breeding has elucidated organ development of foxtail millet spikelets. Zhang et al. (2017) constructed an F2 mapping population by crossing narrow spikelet mutants with normal-type strains and using BSA-Seq position cloning technology and SSR molecular labeling. Thus, they localized the narrow spikelet mutant gene sins1 within the 7.709 Mb region between markers 3-2658 and CAAS3031. Xiang et al. (2017) examined foxtail millet loose-panicle mutants, showing that mutation of the Seita.5G387200 gene on chromosome II resulted in early termination of protein translation, leaving the siaux1 mutant with both lax primary branching pattern, low selfing, and floret fertility. Xue et al. (2018) identified two genes, Seita.1G106700 and Seita.1G107200, through phenotypic analysis and gene mapping of the spike tip abortion mutant sipaa1 associated with undeveloped or incomplete development of foxtail millet, demonstrating that spike tip abortion of foxtail millet may be caused by programmed cell death.

In our study, two populations with extreme traits associated with foxtail millet spikelet development were constructed by hybridization for BSA-Seq analysis. It was preliminarily determined that the three candidate regions on chromosomes III, VI, and VII and 90 genes with non-synonymous mutations in these three regions of the foxtail millet genome might be related to spikelet development. Nevertheless, no previous studies on the development of spikelet organs in these three candidate regions have been found (Wei et al., 2021). By analyzing the developmental transcription process of foxtail millet floral organs by BSR-Seq, more than 2,000 DEGs were identified between foxtail millet with spikelet sterility and normal-developing foxtail millet. This also showed that the infertility of foxtail millet spikelets may be caused by the mutual action of multiple genes. However, combined with segregation of the trait in the F2 population, it appears that the occurrence of this phenotype was probably affected by a pair of recessive main effect genes, and thus, changes in other genes may occur under the regulation of this locus. Through the combined analysis of the results of BSA-Seq and BSR-Seq, we obtained six candidate genes with different structures and functions. Among them were beta-glucosidase 12, non-specific serine/threonine protein kinase, FBD domain-containing protein, sugar transport protein MST1, VQ motif-containing protein 11, and ETHYLENE INSENSITIVE 3-like 5 protein, and proteins involved in carbohydrate metabolism, pollen recognition, WRKY transcription factor activity regulation, monosaccharide transmembrane transport, ethylene reaction pathway, floral meristem, and floral organ morphogenesis.

Among the candidate genes, monosaccharides and sucrose transporters play a pivotal role in saccharide transport from source to sink (Smeekens, 2000). Studies on transgenic tobacco indicate that monosaccharide transporters may play an important role in photosynthate transport and morphogenesis (Leterrier et al., 2003). In rice, members of different monosaccharide transporter families often have different expression patterns. Expression of OsMST5 is only strongly detected in panicles before heading and has been suggested to be associated with pollen development (Ngampanya et al., 2003). Additionally, OsMST8 and OsMST4 are also actively involved in rice anther development (Wang et al., 2007). F-box motifs are widely present in protein interactions, and F-box proteins are involved in the regulation of various development processes such as plant photomorphogenesis, circadian clock regulation, self-incompatibility, floral meristem development, and floral organ identity determination (Hong et al., 2012). In *Arabidopsis*, the F-box protein COI1 recruits regulatory factors of defense response and pollen development through ubiquitination modification, while another F-box protein, UFO, regulates flower organ recognition by activating or maintaining the transcription of the class B organ recognition gene APETALA3 (Xie et al., 1998; Samach et al., 2010). The VQ motif-containing protein family is a plant-specific transcription regulation cofactor, which not only plays an important role in the regulation of plant growth, development, and responses to various external environmental stresses but also participates in regulating the growth and development of seed, hypocotyl, flower, and leaf tissues (Zhang and Wei, 2019). A study in *Arabidopsis* has shown that VQ proteins, as cofactors interacting with transcription factors (e.g., WRKY, MAPK), can usually interact with transcription factors or themselves to regulate various physiological and biochemical processes in plants (Cheng et al., 2012). Ethylene is a simple gas molecule with biological activity, which regulates the growth of plants and many physiological processes, such as seed germination, root hair development, flowering, fruit ripening, organ senescence, and plant responses to biotic or abiotic stress (Bleecker and Kende, 2000). Ethylene-insensitive3 (EIN3) is a transcription factor, and its main target is ethylene response factor1 (ERF1), which is an early ethylene response gene encoding GCC-box-binding protein (Kosugi and Ohashi, 2000). Additionally, EIN3 protein acts upstream of ERF1 and specifically binds to a palindromic repeat in the ERF promoter to regulate a late ethylene response gene containing GCC-box in its promoter (Song et al., 2015).

As a reaction catalyzed by protein kinases, protein phosphorylation is one of the most important post-translational protein modifications, and it is related to the regulation of many biological activities and functions in cell signaling. When plants encounter stress, trauma, or stimulation by other cytokines and hormones, serine/threonine protein kinase is rapidly phosphorylated and activated at serine and threonine residues. It further activates downstream signal molecules through cascade phosphorylation, activates specific signal transduction pathways, and finally transmits external signals to the nucleus to activate or inhibit the expression of specific genes (Hasegawa et al., 2000; Afzal et al., 2008; Zipfel, 2008).

Based on the functional analysis of the above genes and the published literature, we speculated that the six candidate genes in Table 4 are involved in the development of foxtail millet spikelet. Further analysis combined with RT-qPCR experimental results revealed that Setit009293mg exhibited the greatest change compared with other candidate genes between the different fertility samples, and GO enrichment analysis also showed Setit009293mg was mainly enriched in the processes of pollination and pollen-pistil interaction. Accordingly, we speculated that Setit009293mg was the most critical of the six candidate genes ultimately related to fertility. However, the development of millet spikelets is a complex process, which involves complex regulatory networks and multiple metabolic pathways (Wang et al., 2020; Shen et al., 2021). The occurrence of foxtail millet spikelet sterility might have been caused by non-synonymous mutations in the above gene located upstream, which led to the change of gene function, thus triggering a series of changes in downstream genes. The upregulation of Setit009293mg was associated with sterility, which affected the substrate of an rRNA methyltransferase activity-related gene (Setit011746mg), resulting in downregulation of its expression. However, owing to the occurrence of sterility in millet, the normal process from grain filling to maturity cannot be completed, resulting in a decrease in the expression of a gene regulating ethylene (Setit009909mg). At the same time, a gene related to the negative regulation of carbohydrate synthesis and thus related to the accumulation of organic matter in the growing process increased significantly (Setit009896mg), and a gene related to monosaccharide transport decreased significantly (Setit009865mg). The decrease of Setit011801mg expression in sterile samples may be a regulatory mechanism in the process of abiotic stress (Kim et al., 2013).

**TABLE 4:** The final candidate genes related to spikelet development.

Gene ID	Protein ID	FPKM		Description
		BY	KY	
Setit009896mg	K3Y6Q5	1.81	0.77	Glucosidase activity
Setit009293mg	K3Y505	1.38	0.24	Protein phosphorylation
Setit011746mg	K3YC02	0.29	0.62	Serine/threonine-protein phosphatase PP2A-1 catalytic subunit
Setit009865mg	K3Y6M4	2.21	6.54	Secondary carrier transporter
Setit011801mg	K3YC57	2.38	8.52	VQ motif-containing protein 11-like
Setit009909mg	K3Y6R8	1.15	9.71	DNA-binding transcription factor Activity transcription regulatory region sequence-specific DNA binding

*FPKM: the number of genes expressed after FPKM standardization.*

Through the combination of BSA-Seq and BSR-Seq, candidate genes related to millet spikelet development were preliminarily identified, and their functions and possible related pathways were preliminarily examined and confirmed by RT-qPCR. This study provides a reference for the improvement of millet yield and the study of yield-related traits in other gramineous crops. However, there are still some limitations in the functional verification and mechanism of action of genes in the experiment. In future research, we will continue to verify and explore the detailed mechanism of foxtail millet sterility based on the identified candidate genes.

## Conclusion

Three regions associated with abnormal floret development were identified on chromosomes III, VI, and VII by BSA-Seq, and 90 candidate genes were identified. Combined with BSR-Seq results and biological analysis, six candidate genes related to foxtail millet sterility were finally determined, and Setit009293mg was considered to be the main gene controlling the sterility phenotype. This study lays a foundation for future breeding and map-based cloning of related genes in foxtail millet and other C_4_ crops.

## Data Availability Statement

The original contributions presented in the study are publicly available. This data can be found here: PRJNA792301

## Author Contributions

YG: methodology, formal analysis, visualization, writing—review, and editing. LD: conceptualization and resources. QM: investigation and data curation. YY: validation, writing—review, and editing. JL: methodology and data curation. HS and BF: supervision and funding acquisition. All authors: contributed to the article and approved the submitted version.

## Conflict of Interest

The authors declare that the research was conducted in the absence of any commercial or financial relationships that could be construed as a potential conflict of interest.

## Publisher’s Note

All claims expressed in this article are solely those of the authors and do not necessarily represent those of their affiliated organizations, or those of the publisher, the editors and the reviewers. Any product that may be evaluated in this article, or claim that may be made by its manufacturer, is not guaranteed or endorsed by the publisher.

## Footnotes

1
http://www.geneontology.org/

2
http://www.kegg.jp
